# Classification of glioma based on prognostic alternative splicing

**DOI:** 10.1186/s12920-019-0603-7

**Published:** 2019-11-15

**Authors:** Yaomin Li, Zhonglu Ren, Yuping Peng, Kaishu Li, Xiran Wang, Guanglong Huang, Songtao Qi, Yawei Liu

**Affiliations:** 1 Department of Neurosurgery, Nanfang Hospital, Southern Medical University, Guangzhou, Dadao Bei Street 1838#, Guangzhou, People’s Republic of China; 2 Laboratory for Precision Neurosurgery, Nanfang hospital, Southern Medical University, Guangzhou, 510515 Guangdong China; 3 College of Medical Information Engineering, Guangdong Pharmaceutical University, Guangzhou, 510515 Guangdong China

**Keywords:** Glioma, Glioblastoma, Alternative splicing, Prognosis, Classification

## Abstract

**Background:**

Previously developed classifications of glioma have provided enormous advantages for the diagnosis and treatment of glioma. Although the role of alternative splicing (AS) in cancer, especially in glioma, has been validated, a comprehensive analysis of AS in glioma has not yet been conducted. In this study, we aimed at classifying glioma based on prognostic AS.

**Methods:**

Using the TCGA glioblastoma (GBM) and low-grade glioma (LGG) datasets, we analyzed prognostic splicing events. Consensus clustering analysis was conducted to classified glioma samples and correlation analysis was conducted to characterize regulatory network of splicing factors and splicing events.

**Results:**

We analyzed prognostic splicing events and proposed novel splicing classifications across pan-glioma samples (labeled pST1–7) and across GBM samples (labeled ST1–3). Distinct splicing profiles between GBM and LGG were observed, and the primary discriminator for the pan-glioma splicing classification was tumor grade. Subtype-specific splicing events were identified; one example is AS of zinc finger proteins, which is involved in glioma prognosis. Furthermore, correlation analysis of splicing factors and splicing events identified SNRPB and CELF2 as hub splicing factors that upregulated and downregulated oncogenic AS, respectively.

**Conclusion:**

A comprehensive analysis of AS in glioma was conducted in this study, shedding new light on glioma heterogeneity and providing new insights into glioma diagnosis and treatment.

## Background

Glioma classification based on molecular characteristics plays an increasingly important role in diagnosis and treatment of glioma. Genetic, DNA methylation, gene expression features have been demonstrated to influence the prognosis of glioma patients [1, 2], and related molecular signatures, such as isocitrate dehydrogenase genes 1 and 2 (IDH1/IDH2) status, O^6^-methylguanine-DNA methyltransferase (MGMT) promotor status, codeletion of chromosome arm 1p and 19q (1p/19q codel) and TERT promoter status have been applied widely in prognosis prediction [3]. Previous research on glioma classification has assisted in the prediction of prognosis and the guidance of treatment regimens, for example, the transcriptional classification of glioma reported by The Cancer Genome Atlas (TCGA) defined 4 subtypes, classical (CL), mesenchymal (MES), neural (NE), and proneural (PN), advancing our knowledge for the improvement of glioma diagnosis and therapy [1, 4–6]. However, glioma remains a serious threat to patients, especially the most aggressive kind, glioblastoma (GBM). Due to the lack of an effective treatment, the median survival of GBM patients is only 14.6 months after current standard therapy [7]. We focused on a novel approach, classification of glioma based on alternative splicing (AS) event profiles, to understand glioma more comprehensively and explore new ideas for its diagnosis and treatment.

AS is a dynamic process that occurs after a gene is transcribed into precursor mRNA, and leads to complexity of the transcriptome. Changes in splicing patterns affect protein structure and function.

AS plays critical roles in oncogenesis. Cancer-specific mRNA transcripts, may result in loss-of-function of tumor suppressors or activation of oncogenes and cancer pathways [8]. Most recently, a study comprehensively analyzed different tumor types from TCGA datasets to detect tumor-specific AS in combination with proteomic analysis; this study proposed a new method for exploring peptides as potential antigens in tumor immunotherapy [9]. In addition to identifying tumor-specific AS, the biological effects of AS on cancer progression, especially prognosis-related AS, also warrant attention. Comprehensively analysis of non-small cell lung cancer [10], colorectal cancer [11] and esophageal carcinoma [12] revealed the prognostic significance of AS. AS of ANXA7, MARK4, MAX, USP5, WWOX, BIN, RON, and CCND1 were reported to affect critical biological functions of glioma, resulting in altered prognosis [13–15]; however, a systematic analysis of glioma splicing profiles has not been performed. Building on the availability of RNA-seq data of TCGA, we performed a comprehensive analysis of splicing events associated with prognosis in patients with glioma. Moreover, based on prognosis-related splicing events, we identified novel classification of glioma with distinct splicing characteristics and clinical features, shedding light on the new ideas for glioma research.

## Methods

### Data acquisition

RNA-seq data and corresponding clinical data for 665 glioma samples (154 GBM, 511 LGG) were acquired from the data portal for TCGA (https://portal.gdc.cancer.gov/;DbGaP Study Accession:phs000178), using R/Bioconductor package TCGAbiolinks. Percent Spliced In (PSI) values, the percentage of splicing events in the abovementioned samples, were downloaded by using TCGASpliceSeq [16] (http://bioinformatics.mdanderson.org/TCGASpliceSeq), a web-based platform that provides splicing patterns of TCGA tumors. In this platform, splicing events were divided into 7 categories, including Exon Skip (ES), Retained Intron (RI), Alternate Promoter (AP), Alternate Terminator (AT), Alternate Donor site (AD), Alternate Acceptor site (AA), and Mutually Exclusive Exons (ME). In total, 83,776 splicing events were detected in GBM dataset, including 47,672 ES, 3574 RI, 12425 AP, 9210 AT, 4910 AD, 5630 AA and 355 ME. A total of 96,419 splicing events were detected in the LGG dataset, including 58,503 ES, 3694 RI, 13183 AP, 9366 AT, 5260 AD, 5954 AA and 458 ME.

### Survival analysis

The patients were divided into two groups by the mean cutoff PSI for each splicing event. To conduct further clustering analysis, which was needed to ensure that the data were not null and exclude the splicing events affected by outliers, splicing events that met the following conditions were included: 1) the PSI value was not missing for any samples; and 2) the sample size of each group was higher than 5% of the total size (*n* ≥ 8 in GBM, *n* ≥ 33 in glioma). Overall, 16,173 events across the pan-glioma samples and 20,939 events across the GBM samples matched. The log-rank test was used to assess the relationship between overall survival (OS) and AS events. Splicing events with a *p*-value< 0.01 in the log-rank test were regarded as prognostic. The same strategy was performed in additional studies, including survival analysis for splicing subgroups and splicing factors, as described in our former study [17–19].

### Clustering analysis

Clustering analysis of prognosis-related splicing events was performed across all glioma (GBM + LGG) samples and for GBM samples.

Monte Carlo Consensus Clustering (M3C), a consensus clustering-based algorithm with a hypothesis testing framework, was used in our clustering study. The R package of M3C was downloaded from Bioconductor (https://www.bioconductor.org/). To evaluate the best number of subgroups (k), we comprehensively considered the following indexes: cumulative distribution function (CDF), empirical *p* value, proportion of ambiguous clustering (PAC) and relative cluster stability index (RCSI).

### DNA methylation profiling

We evaluated the DNA methylation levels across splicing clusters by using a strategy similar to that reported by Michele Ceccarelli did [2].

### Subtype-specific signatures

Splicing events in each subtype were regarded as signatures if their mean PSI values were 30% higher than those in any other subtype (FDR < 0.05). Statistical analysis was performed by using the Mann-Whitney U test. The R package Scales was used to convert the PSI values of each splicing event to a scale from 0 to 1; subsequently, the converted values were displayed in heatmaps.

### Correlation analysis of splicing factors and splicing events

Splicing factors with recognized prognostic significance in cancers, especially in glioma and pST1-specific splicing events, were selected for the analysis. A Spearman correlation test was used to evaluate the association between expression levels of splicing factors and PSI values of pST1-specific splicing events. Correlations with a coefficient > 0.45 and *p*-value< 0.05 were considered to be significant and are shown in the network diagram. Cytoscape (version 3.5.1) was used to construct the network of relationships.

### IDH-status-related splicing events

Prognostic splicing events with mean PSI values that exceeded twice the difference between groups were regarded as IDH status-related splicing events (FDR < 0.05). Statistical analysis was performed by using the Mann-Whitney U test. The R package Scales was used to convert the PSI values of each splicing event to a scale from 0 to 1; subsequently, the converted values were displayed in heatmaps.

## Results

### Identification of prognostic alternative splicing events in the TCGA GBM and LGG datasets

**Fig. 1:**
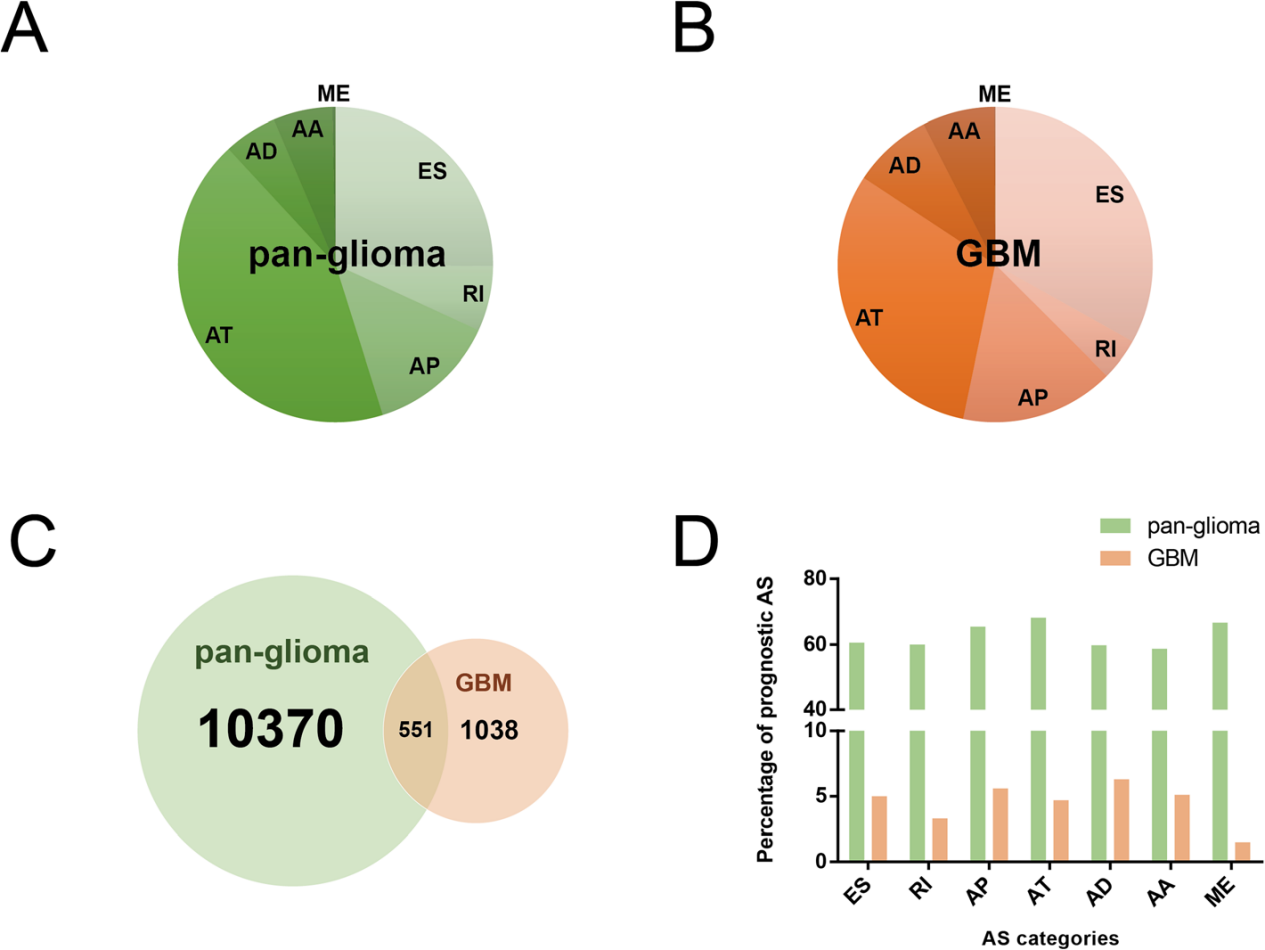
Analysis of prognostic splicing events in pan-glioma/GBM samples. **a** The distribution of alternative splicing categories of prognostic splicing events in pan-glioma samples. **b** The distribution of alternative splicing categories of prognostic splicing events in GBM samples. **c** Venn diagrams showing the distribution of prognostic splicing events in pan-glioma/GBM samples. **d** Percentage of prognostic alternative splicing events in 7 splicing categories

Splicing event profiles were analyzed in depth for 154 GBM patients and 511 LGG patients from TCGA (https://portal.gdc.cancer.gov/;DbGaP Study Accession:phs000178). Univariate survival tests were conducted to assess the correlation between the PSI of splicing events and OS (Additional file 1: Table S1, Additional file 2: Table S2). First, to search for prognostic splicing events in the pan-glioma samples, we combined the GBM and LGG datasets and conducted a comprehensive survival analysis; we identified 10,370 splicing events with prognostic significance, including 2611 ES, 693 RI, 1378 AP, 4456 AT, 568 AD, 632 AA and 32 ME (Fig. 1a, Additional file 1: Table S1). Further analysis revealed that there is a substantial difference in splicing patterns between GBM and LGG. Survival analysis was then performed for GBM alone. A total of 1038 splicing events were subsequently detected as significantly associated with GBM patient survival, including 344 ES, 45 RI, 164 AP, 322 AT, 85 AD, 77 AA and 1 ME (Fig. 1b, Additional file 2: Table S2). A total of 551 splicing events were involved in both pan-glioma and GBM prognosis (Fig. 1c). Interestingly, over 50% of splicing events were prognostic in each splicing category across pan-glioma samples, while less than 7% of splicing events were prognostic in each splicing category across GBM samples (Fig. 1d).

### Consensus clustering identifies seven splicing types of glioma

**Fig. 2:**
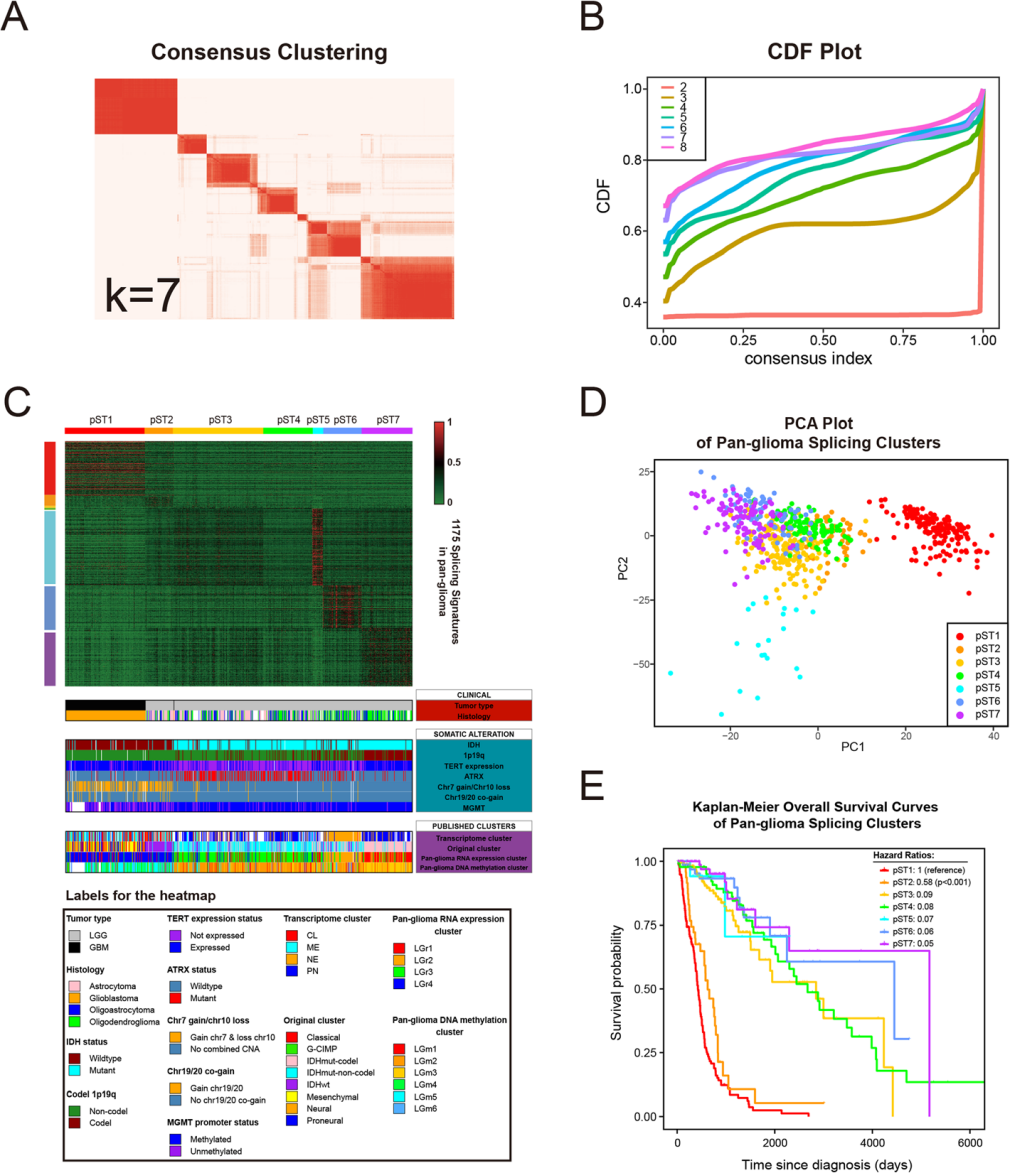
Identification of 7 splicing types of pan-glioma samples. **a** Consensus clustering matrix of 665 glioma samples for k = 7. **b** CDF plot for k = 2 to k = 8. **c** Heatmap of percent spliced in (PSI) values. Columns represent 7 splicing types across 665 TCGA glioma samples, labeled pST1–7; rows represent PSI values of splicing signatures in pST1–7. **d** Principal component analysis (PCA) of pST1–7. **e** Kaplan-Meier overall survival curves for pST1–7

To segregate the splicing subtypes across the pan-glioma samples, we performed M3C analysis for prognosis-related splicing events with 665 glioma samples. The data showed that the clustering stability was best at k = 2 or 7 (Fig. 2a, b, Additional file 7: Figure S1). Regardless of whether k = 2 or k = 7, glioma grade was the principal discriminator for GBM and LGG samples that were notably distributed in different clusters, demonstrating that clustering based on our criteria is consistent with the clinical classification of gliomas and indicating that splicing profiles differ between GBM and LGG (Fig. 2c). To explore the intrinsic characteristics of LGG, we further analyzed glioma splicing types at k = 7, labeled pST (pan-glioma Splicing Type) 1–7, which contained 153, 54, 173, 94, 20, 74, and 97 samples, respectively. Consistent with previous data, principal component analysis (PCA) based on signatures of pST1–7 in further analysis also revealed marked differences between GBM and LGG samples (Fig. 2d). As previously mentioned, pST1 was exclusively from the GBM dataset and included nearly all GBM samples (153/154, 99.4%) (Table 1). The other six clusters are almost all from LGG, except for one pST3 sample from GBM (Table 1).

**Table 1:** Clinical characteristics of pan-glioma splicing types

	pST1(*n* = 153)	pST2(*n* = 54)	pST3(*n* = 173)	pST4(*n* = 94)	pST5(*n* = 20)	pST6(*n* = 74)	pST7(*n* = 97)	Total(*n* = 154)
Clinical								
Age								
Median	60	57	38	38	50	40	43.5	47
Survival (in months)								
Median (CI)	13.5 (12.1,15.3)	20.2 (17.9, 27.1)	94.5 (55.5, NA)	88.7 (68.4132.6)	NA (32.1, NA)	148.2 (74.5, NA)	172.2 (76.1, NA)	51.2 (44.6, 66.7)
Karnofsky score								
100	12	4	30	17	2	4	17	86
90	2	12	41	23	3	13	19	113
70–80	69	11	15	10	3	12	10	130
< 70	31	3	6	3	0	1	3	47
Sex								
Female	54	26	53	38	9	35	38	253
Male	99	27	85	56	7	35	46	355
Grade								
G2	0	4	70	44	7	45	44	214
G3	0	49	67	50	9	25	40	240
G4	153	0	1	0	0	0	0	154
Histology								
Astrocytoma	0	35	68	37	7	15	7	169
Glioblastoma	153	0	1	0	0	0	0	154
Oligoastrocytoma	0	9	39	25	3	18	19	113
Oligodendroglioma	0	9	30	32	6	37	58	172
Molecular								
IDH Status								
Mutant	9	5	151	88	17	59	96	425
WT	140	49	21	5	3	14	1	233
1P19Q								
Codel	0	1	23	20	9	28	85	166
Non-codel	147	53	150	74	11	46	12	493
TERT Expression Status								
Expressed	127	39	40	20	12	27	82	347
Not.expressed	25	15	132	73	8	46	15	314
ATRX Status								
Mutant	8	6	94	53	4	21	9	195
WT	138	48	78	40	16	52	88	460
CHR7.Gain.CHR10.loss								
Gain.chr7&loss.chr10	97	37	14	0	1	2	0	151
No.combined. CNA	50	17	159	93	19	70	97	505
CHR19.20.co.gain								
Gain.chr19/20	19	6	3	0	0	2	0	30
No.chr19/20.gain	128	48	170	93	20	70	97	626
MGMT Promoter								
Methylated	51	25	147	78	16	63	94	474
Unmethylated	71	29	26	16	4	11	3	160
Clusters								
Transcriptome Cluster								
CL	49	26	7	1	1	1	0	85
MC	66	16	8	6	1	0	0	97
NE	5	1	19	9	0	64	12	110
PN	18	3	72	61	7	4	69	234
Original Cluster								
Classical	39	0	0	0	0	0	0	39
G-CIMP	7	0	1	0	0	0	0	8
IDHmut-codel	0	1	23	20	9	28	85	166
IDHmut-non-codel	0	4	127	67	8	31	11	248
IDHwt	0	49	21	6	3	14	1	94
Mesenchymal	50	0	0	0	0	0	0	50
Neural	26	0	0	0	0	0	0	26
Proneural	29	0	0	0	0	0	0	29
Pan-Glioma RNA Expression Cluster								
LGr1	20	4	25	17	9	0	64	139
LGr2	3	0	0	2	0	63	21	89
LGr3	9	4	127	68	10	7	12	237
LGr4	121	46	20	6	1	3	0	197
Pan-Glioma DNA Methylation Cluster								
LGm1	7	4	18	11	2	7	3	52
LGm2	1	0	120	59	7	38	27	252
LGm3	0	1	14	19	8	14	66	122
LGm4	43	14	4	0	1	3	0	65
LGm5	59	30	12	0	1	2	0	104
LGm6	12	5	5	5	1	10	1	39

To identify the clinical features and molecular characteristics of each cluster, we analyzed clinical data of each sample from TCGA and identified crucial molecular indicators of glioma. DNA methylation profiles were also shown to be clinically relevant for glioma classification [2]. To determine the relationship between DNA methylation profiles and AS profiles, we performed an integrated analysis of data from TCGASpliceSeq and the TCGA methylation platform, including the HumanMethylation450/27 platforms.

**Fig. 3:**
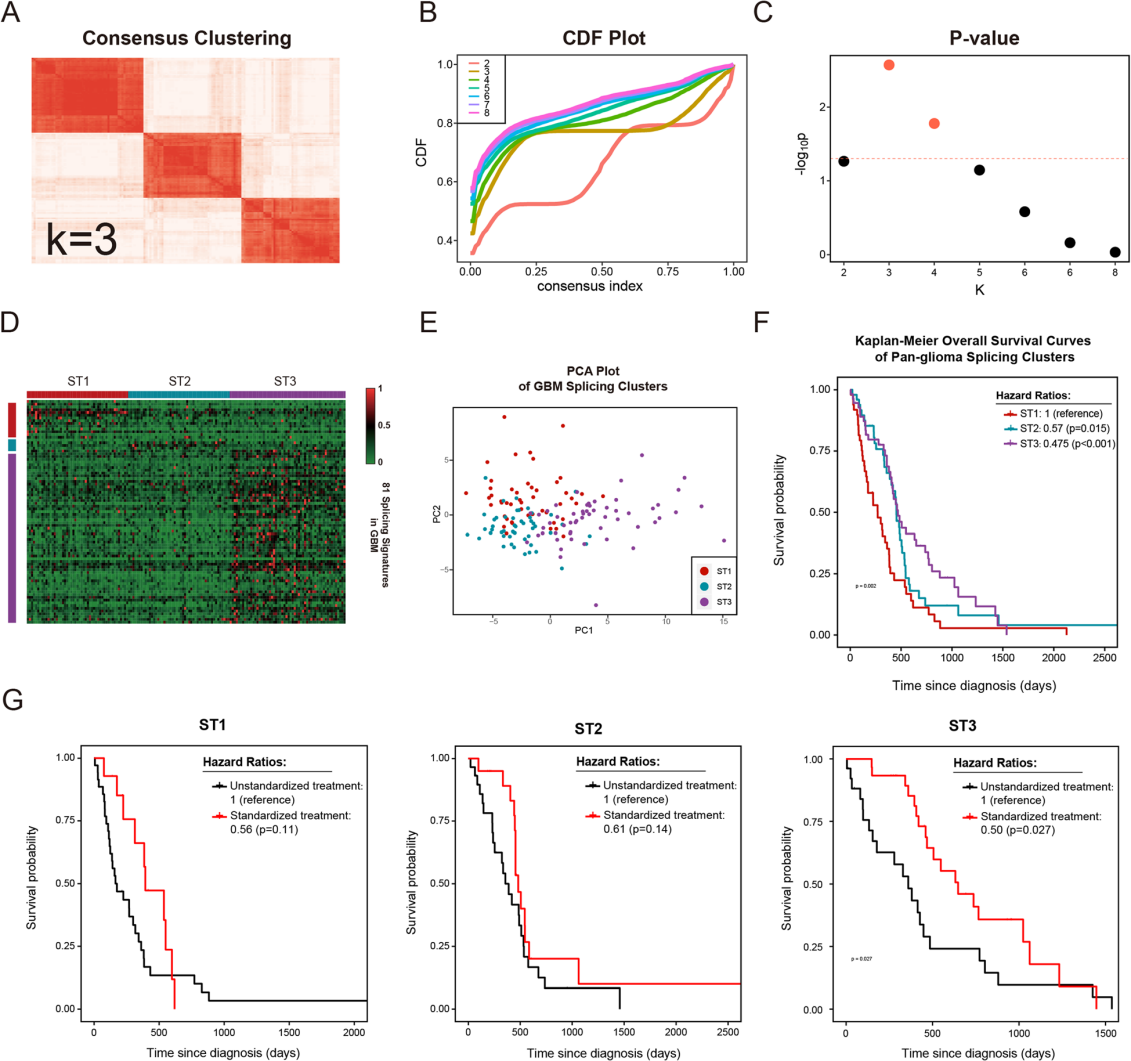
Identification of 3 splicing types of GBM samples. **a** Consensus clustering matrix of 154 GBM samples for k = 3. **b** CDF plot for k = 2 to k = 8. **c** *P*-values for k = 2 to k = 8. **d** Heatmap of percent spliced in (PSI) values data. Columns represent 3 splicing types across 154 TCGA GBM samples, labeled ST1–3; rows represent PSI values of splicing signatures in ST1–3. **e** Principal component analysis (PCA) of ST1–3. **f** Kaplan-Meier overall survival curves of ST1–3. **g** Kaplan-Meier overall survival curves of patients classified by therapy regimen of ST1–3

Surprisingly, pST2–7 had distinct clinical and molecular features; among those types, the characteristics of pST2 were remarkable. Compared to pST3–7, pST2 contains a higher proportion of grade III gliomas (49/53, 92.5%, *p* < 0.01) and a higher proportion of astrocytoma (35/53, 66.0%, p < 0.01) (Table 1). Clinically, pST2 resembled pST1 or GBM, with a higher diagnosis age and an unfavorable prognosis (Table 1 and Fig. 2e). Interestingly, pST2 also shared characteristics similar to those of pST1 or GBM, with molecular enrichment for IDH wildtype (49/54, 90.7%, *p* < 0.01), 1p/19q non-codeletion (53/54, 98.1%, p < 0.01), MGMT promotor unmethylated (29/54, 53.7%, p < 0.01), chromosome 7 gain paired with chromosome 10 loss (37/54, 68.5%, p < 0.01), chromosome 19/20 gain (6/54, 11.1%, p < 0.01) (Table 1 and Fig. 3c). In addition, compared to pST3–7, pST1–2 showed genome-wide hypomethylation (Additional file 9: Figure S3). Overall, although the pST2 samples were all from the LGG dataset, this group had a malignant phenotype similar to that of GBM. Unlike pST2, pST7 harbored oligodendroglioma (58/84, 69.0%, *p* < 0.01), as indicated by histological analysis (Table 1 and Fig. 3c). Moreover, patients in pST7 had better clinical outcome, with molecular phenotypes characteristic of a favorable prognosis. Notably, pST7 was enriched for IDH mutation (96/97, 99.0%, *p* < 0.01), 1p/19q co-deletion (85/97, 87.6%, p < 0.01), and MGMT promotor methylation (94/97, 96.9%, p < 0.01), and it harbored no chromosome 7 gain paired with chromosome 10 loss or chromosome 19/20 gain (Table 1 and Fig. 3c).

Subsequently, we found our novel splicing clusters correlated to the published classifications of glioma, taking transcriptome subtype (CL, MES, NE, PN) for example, most of the pST1–2 samples were CL or MES (157/184, 85.3%), while pST6 were mainly NE (64/69, 92.8%) and pST3, 4, 5, 7 were mainly PN (p < 0.01) (Table 1 and Fig. 3c). Meanwhile, comparing to DNA methylation subtypes (LGm1–6), clear tendency of the distribution was observed: 92.6% (163/176) pST1–2 samples were divided into LGm4–6; while 90.2% (413/458) pST3–7 samples were divided into LGm1–3, which is genome-wide hyper-methylation (*p* < 0.01) (Table 1 and Fig. 3c).

Glioma classification analysis of genomics, RNA expression profiling, epigenetics or proteomics had been published. In this study, classification based on AS was also conducted. Actually, correlation among classifications were common [2], indicating that clinical phenomenon could be related to multiple molecular characteristics of different levels. Using these classifications comprehensively might be more reliable.

### Consensus clustering identifies three splicing types of GBM

Compared to LGG, GBM has a distinct genotype and phenotype and a different splicing profile, according to our data. In addition, GBM requires more aggressive treatment due to its unfavorable outcomes and refractoriness. Heterogeneity in GBM is considered to contribute to treatment resistance [20, 21]. By using the single-cell RNA-seq technique, which was developed rapidly in recent years, intratumoral heterogeneity in GBM was identified at the single-cell level [22, 23]. To reveal the heterogeneity in GBM and offer more targeted suggestions for GBM diagnosis and treatment, classification of GBM according to molecular phenotype has always been the standard method [1, 2]. However, classification for GBM based on AS has not yet been explored. Using the same strategy used for pan-glioma classification, we performed a classification study for GBM alone. Monte Carlo Consensus Clustering of prognosis-related splicing profiles for 154 GBM samples identified three robust clusters (Fig. 3a, d), labeled ST (splicing type) 1–3, with the flattest curve in the CDF plot (Fig. 3b), the lowest *p*-value (Fig. 3c), and elbows in the PAC score curve and RCSI curve (Additional file 8: Figure S2A, S2B) at k = 3. ST1, ST2 and ST3 contain 49, 49 and 56 samples, respectively. PCA based on signatures of ST1–3 was also performed in further analysis to show the differences among the 3 splicing types of GBM samples (Fig. 3e). To identify the molecular characteristics of each cluster, we also analyzed several recognized molecular indicators for GBM, including IDH status, CpG island methylator phenotype (CIMP), and MGMT promoter status. In total, 6.8% (10/146) IDH mutations were observed in all GBM samples and differed among the three splicing clusters. IDH mutations occurred most frequently in ST3 (7/56, 12.5%), with no IDH mutations in ST1 (0/46) and a frequency of 6.3% (3/48) in ST2 (*p* < 0.05) (Table 2). Consistent with the association between IDH status and CIMP in glioma, ST1 was exclusively non-G-CIMP (48/48, 100%), while G-CIMP samples were enriched in ST2 (2/48, 4.2%) and ST3 (6/56, 10.7%) (p < 0.05) (Table 2). MGMT promoter status also differed among the three clusters. A higher proportion of MGMT promoter methylation samples was observed in ST3 (24/45, 53.3%) than in ST1 (11/37, 29.7%) or ST2 (17/41, 41.5%) (*p* = 0.099) (Table 2).

**Table 2:** Clinical characteristics of GBM splicing types

	ST1(*n* = 49)	ST2(n = 49)	ST3(*n* = 56)	Total(n = 154)
Clinical				
Age				
Median	62.8	60.1	61.0	60.24
No. ≤ 40 years old	1	5	6	12
Survival (in months)				
Median (CI)	9.0 (5.47, 12.8)	15.1 (13.5, 17.7)	15.6 (13.5–25.5)	13.8 (12.1–15.3)
Disease-free survival (in months)				
Median (CI)	8.4 (6.0, NA)	8.5 (6.7 13.1)	11 (5.9, NA)	8.5 (6.7, 13)
Karnofsky score				
100	2	2	7	11
90	0	2	0	2
70–80	19	25	19	63
< 70	8	6	16	30
Sex				
Female	18	20	16	54
Male	31	29	40	100
Molecular				
IDH Status				
Mutant	0	3	7	10
WT	46	45	49	140
MGMT Promoter				
Methylated	11	17	24	52
Unmethylated	26	24	21	71
G-CIMP Methylation				
G-CIMP	0	2	6	8
Non-G-CIMP	48	46	50	144
TERT Promoter				
Mutant	4	9	12	25
WT	45	39	41	4
TERT Expression				
Expressed	38	42	47	127
Not.expressed	10	7	9	26
CHR7 Gain CHR10 loss				
Gain chr7&loss chr10	31	32	34	97
No combined CNA	18	13	20	51
CHR19/20 co gain				
Gain chr19/20	2	5	12	19
No chr19/20 gain	47	40	42	129
Clusters				
Transcriptome Cluster				
CL	49	26	7	1
MC	66	16	8	6
NE	5	1	19	9
PN	18	3	72	61
Original Cluster				
Classical	39	0	0	0
G-CIMP	7	0	1	0
IDHmut-codel	0	1	23	20
IDHmut-non-codel	0	4	127	67
IDHwt	0	49	21	6
Mesenchymal	50	0	0	0
Neural	26	0	0	0
Proneural	29	0	0	0
Pan-Glioma RNA Expression Cluster				
LGr1	20	4	25	17
LGr2	3	0	0	2
LGr3	9	4	127	68
LGr4	121	46	20	6
Pan-Glioma DNA Methylation Cluster				
LGm1	7	4	18	11
LGm2	1	0	120	59
LGm3	0	1	14	19
LGm4	43	14	4	0
LGm5	59	30	12	0
LGm6	12	5	5	5

To further clarify the clinical significance of this novel classification based on survival-related splicing events, we analyzed the clinical data of TCGA patients. The most consistent clinical relation for previous GBM transcriptome classification was age, with a markedly higher proportion of younger patients in the proneural classification [1]. In contrast, our GBM splicing subtypes were not significantly related to median age at diagnosis, but the proportion of younger patients (<=40) in ST1 (1/49, 2.0%) was smaller than that in non-ST1 (11/105, 10.5%) (Table 2). Compared to ST2 patients (15.1 months) and ST3 patients (15.6 months), ST1 patients (9.0 months) displayed markedly shorter median survival time (Table 2 and Fig. 3f). Although the difference was not statistically significant, longer disease-free survival was observed in ST3 patients (11.0 months) than in ST1 (8.4 months) and ST2 (8.5 months) patients (Table 2).

To assess the effect of standardized treatment on the 3 clusters, we examined TCGA data and compared the survival of patients receiving standardized treatment, defined as concurrent TMZ chemotherapy and radiotherapy, and nonstandard treatment. Standard treatment significantly improved prognosis in the ST3 cluster (*p* = 0.027), while it did not alter survival in the ST1 and ST2 clusters (Fig. 3g).

Moreover, we compared our classification to the previous transcriptional subtypes of GBM [1], and we found that ST1 samples were mainly distributed in MES (38/48, 79.2%) and rarely distributed in PN (1/48, 2.1%) (*p* < 0.01) (Table 2). Other comparisons of clustering by splicing profiles and clustering by previously reported classifications were also made to show the relationships between these classifications (Table 2).

In summary, clustering analysis of GBM based on prognostic AS events defined 3 subtypes: ST1 has the worst clinical outcome and molecular features of poor prognosis, ST3 has the best clinical outcome, treatment sensitivity and molecular features of favorable prognosis, and ST2 has clinical and molecular characteristics between those of the other 2 subtypes.

### Identification of subtype-specific splicing events

**Fig. 4:**
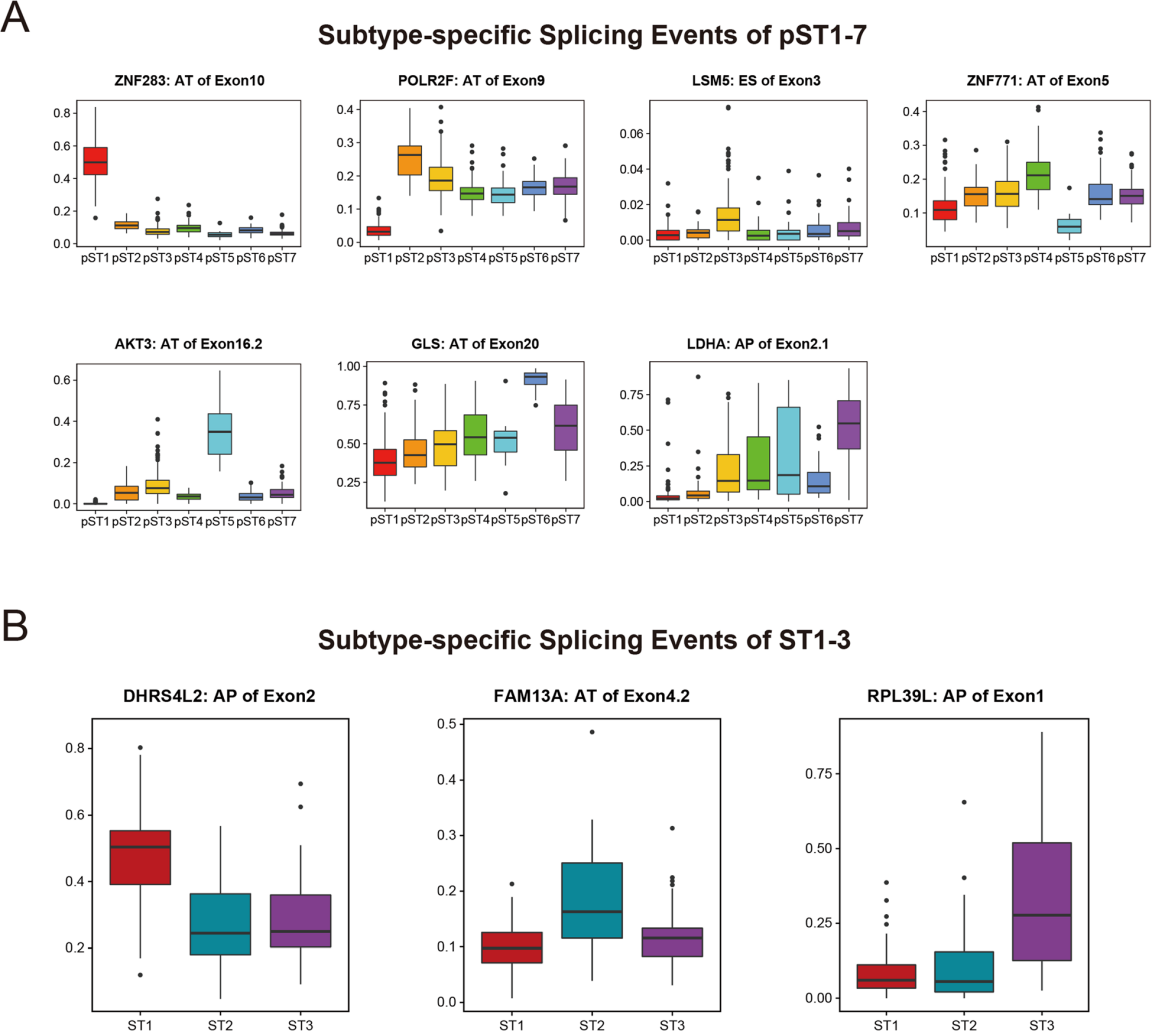
Subtype-specific splicing events of pST1–7 and ST1–3. **a** Representative splicing events of pST1–7; the PSI value of the signature in each cluster was significantly higher than that in any other cluster (*p*-value< 0.05). **b** Representative splicing events of ST1–3; the PSI value of the signature in each cluster was significantly higher than that in any other cluster (*p*-value< 0.05)

To classify external glioma samples, subtype-specific splicing signatures were investigated. The splicing events that increased only in one subtype were identified and defined as signatures of each subtype. A heatmap was generated to display a total of 1175 splicing signatures (pST1–7: 265, 50, 5, 4, 370, 202, and 279) in each subtype of pan-glioma (Fig. 2c and Additional file 3: Table S3). The representative splicing signatures in each cluster were also presented in box plots, including AS of ZNF283 (AT), POLR2F (AT), LSM5 (ES), ZNF771 (AT), AKT3 (AT), GLS (AT), and LDHA (AP) (Fig. 4a). POLR2L, a subunit of RNA polymerase II, was found to be a prognostic splicing factor, and its AS was also prognostic in lung cancer [10]. Here, we also discovered that another subunit of RNA polymerase II, POLR2L, whose AS was an important prognostic signature in pST2, might contribute to the malignant phenotype of low-grade glioma. AS of another splicing factor, LSM5 [24], was specifically upregulated in pST3. AS of AKT3 was reported to play important roles in cancers [25], and AKT3 expression was involved in glioma progression [26, 27]. The data demonstrated that AS of AKT3 was a splicing signature of pST5, which might further reveal the role of AKT3 in glioma. GLS and LDHA are metabolic enzymes that participate in biological progression of IDH-mutant glioma [28, 29]; however, the role of AS of GLS and LDHA in glioma remains unclear. Our data indicated that AS of GLS and LDHA were signatures of pST6 and pST7, respectively, the clusters with the best prognosis, suggesting that both expression and AS of metabolic enzymes might be involved in IDH-mutant glioma.

We performed the same strategy to identify 81 splicing signatures in GBM subtypes (ST1: 14, ST2: 4, ST3: 63) and draw a heatmap (Fig. 3d and Additional file 4: Table S4). Three representative splicing signatures were also shown in box plots (Fig. 4b). DHRS4L2 is a member of the dehydrogenase reductase family [30], with specific alternative transcripts expressed in neuroblastoma [31]. AS of DHRS4L2 might also participate in GBM malignant progression because of its significant increase in ST1. In ST2, AS of FAM13A, a key regulator of lung cancer [32], was discovered as a splicing signature. RPL39L was reported as a ribosomal protein with upregulated expression in cancers and played a role in drug resistance [33–35]. Upregulated AS of RPL39L was observed in ST3 (Fig. 3g), moreover, PSI value of AS of RPL39L was negatively related to mRNA expression level of RPL39L (data not shown). These findings might indicate AS of RPL39L induce downregulation of mRNA expression, leading to sensitivity to concurrent treatment in GBM.

### Regulatory network of splicing factors and pST1-specific splicing events

**Fig. 5:**
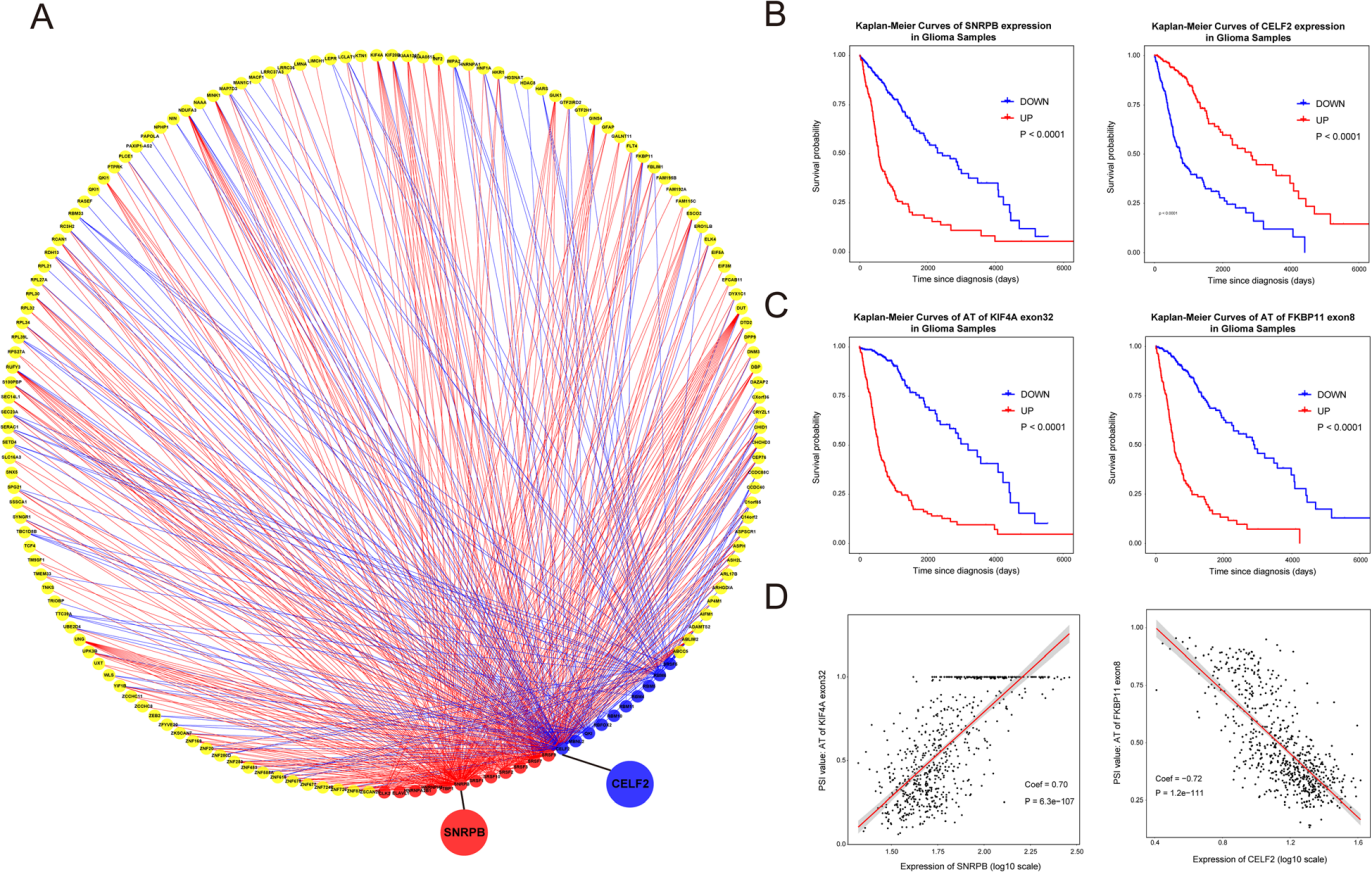
Correlation of splicing factors and pST1-specific splicing events. **a** Regulatory network of splicing factors and pST1-specific splicing events. Yellow nodes represent gene symbols of pST1-specific splicing events. Red nodes represent splicing factors associated with unfavorable prognosis in glioma. Blue nodes represent splicing factors associated with favorable prognosis in glioma. Red/blue lines represent positive/negative correlations. **b** Kaplan-Meier overall survival curves for the expression of SNRPB and CELF2, representative prognostic splicing factors in glioma. **c** Kaplan-Meier overall survival curves for AS of KIF4A and FKBP11, representative prognostic splicing events in glioma. **d** Scatter diagram of the expression of SNRPB and PSI values of AT of KIF4A exon32. Scatter diagram of the expression of CELF2 and PSI values of AT of FKBP11 exon8

Changes in the expression levels of splicing factors significantly affect the development and progression of multiple tumors by regulating AS. Increasing evidence indicates that RNA processing triggered by alterations in splicing factors affects glioma outcomes [36]. To elucidate prognostic AS regulatory processing in glioma, we characterized the regulatory network of pST1-specific splicing events, which represent unfavorable prognostic splicing signatures in glioma. Here, we focused on recognized prognostic splicing factors in tumors, especially in glioma, and identified their prognostic effect on glioma by conducting survival analysis using TCGA RNA-seq expression data. Splicing factors including cancer promoters (CLK2 [37], ELAVL1 [38], HNRNPA2B1 [14], HNRNPH1 [39], PTBP1 [13, 15, 40], SNRPB [41], SRSF1, SRSF2, SRSF3, SRSF7, SRSF9, SRSF10) and cancer suppressors (CELF2, MBLN2, QKI, RBFOX2 [42], RBM4, RBM5, RBM6, RBM10 [43], RBM11 [44], SRSF5) were applied to further correlation analysis. Significant correlations (*p* < 0.05, coefficient > 0.45 or < − 0.45) were shown in the network diagram (Fig. 5a and Additional file 5: Table S5), and 129 pST1-specific splicing events represented by their gene symbols (yellow nodes) are presented. Surprisingly, these pST1-specific splicing events were all positively (red lines) related to the expression of splicing factors associated with unfavorable prognosis (red nodes) and negatively (blue lines) related to the expression of splicing factors associated with favorable prognosis (blue nodes) (Fig. 5a). Meanwhile, we also found that expression level of above-mentioned splicing factors differed among splicing clusters, demonstrating that splicing factors play significant roles in splicing profiles of glioma (Additional file 10: Figure S4).

Recently, research revealed that SNRPB, the key element of spliceosome complex SmB/B′, is an important oncogenic splicing factor in GBM, contributing to the regulation of RNA processing, DNA repair, and chromatin remodeling [45]. Consistent with this finding, the regulatory network of splicing factors and pST1-specific splicing events revealed SNRPB as a hub unfavorable prognostic splicing factor that is positively related to important pST1-specific splicing events (Fig. 5a). CELF2, a suppressor of glioma progression (Fig. 5b), was reported as an important regulator of many AS events across solid cancer samples [46] and exhibited a strong negative correlation with pST1-specific splicing events (Fig. 5a), indicating its key role in glioma suppression via the regulation of numerous splicing events. AS of KIF4A and AS of FKBP11 were hub events in this network (Fig. 5a) with important splicing predictors of glioma prognosis (Fig. 5c). The prominent correlations between expression of SNRPB and AT of KIF4A exon32 (coefficient = 0.70) and between expression of CELF2 and AT of FKBP11 exon8 (coefficient = − 0.72) are shown in scatter diagrams (Fig. 5d).

### Identification of IDH-status-related splicing events

**Fig. 6:**
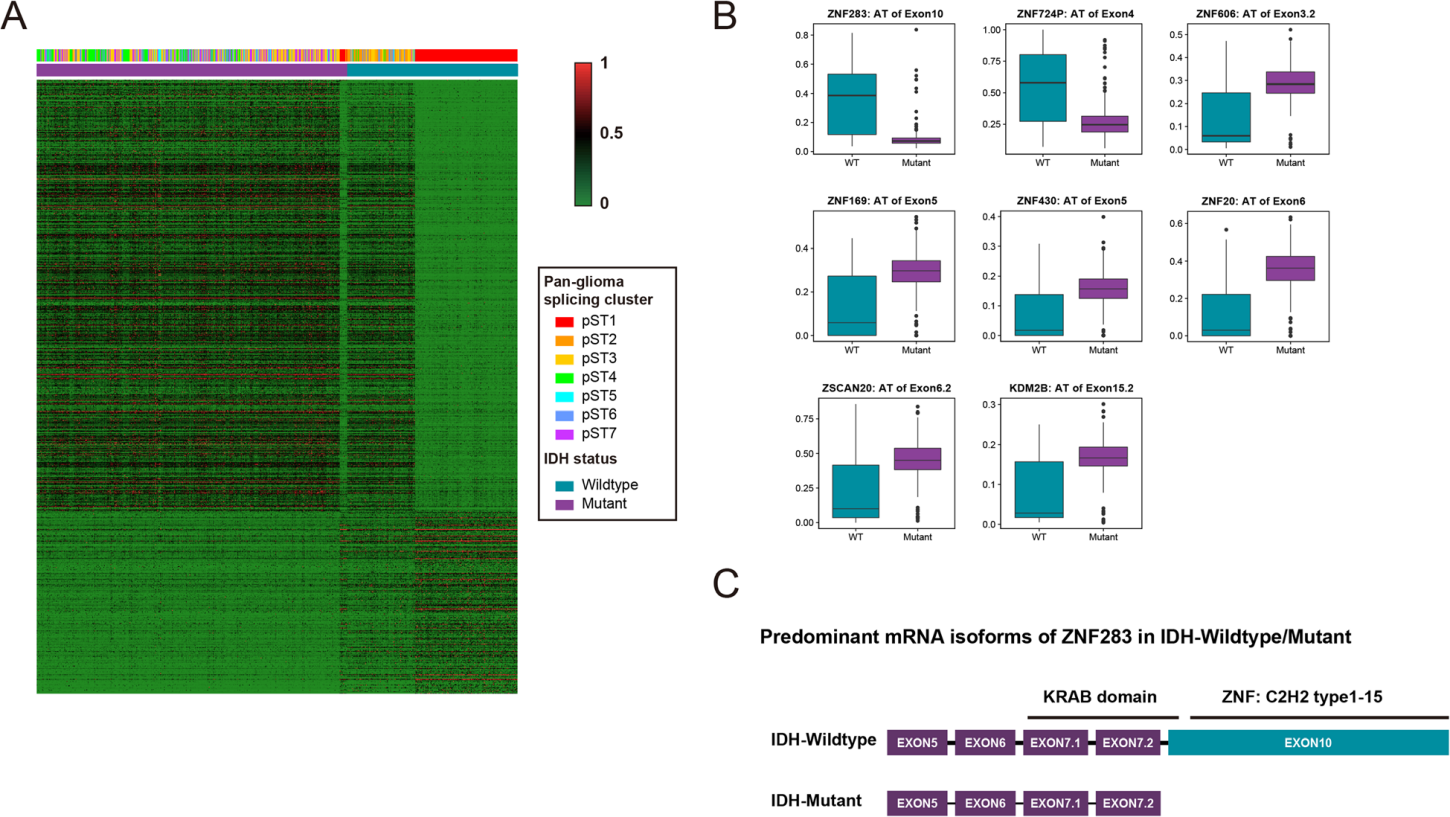
Identification of IDH-status-related splicing events. **a** Heatmap of PSI values. Columns represent IDH-wild type/mutant samples across 658 TCGA glioma samples; rows represent the PSI values of IDH-status-related splicing events. **b** Box plots of 8 representative differential alternative splicing events in zinc finger proteins. **c** Representative sketch of alternative splicing of zinc finger proteins; predominant mRNA isoforms of ZNF283 in IDH-wild type/mutant samples

IDH status is the most significant indicator for prognosis prediction in glioma patients and was the predominant driver of transcriptome/methylome/fCNV glioma classification [2]. In our splicing classification of pan-glioma, in addition to tumor grade, IDH status was also a vital discriminator of splicing clustering. The pST1–2 splicing subgroups harbored IDH-wild type samples (189/203, 93.1%), while pST3–7 harbored IDH mutations (411/455, 90.3%). To identify the IDH status-related prognostic splicing events, we separated the samples into IDH-wild type/mutant groups. Splicing events with mean PSI values that exceeded twice the difference between groups were chosen (FDR < 0.05), and a total of 840 splicing events were observed (Fig. 6a and Additional file 6: Table S6).

Although proteomics research indicated that a large number of genes have a predominant protein isoform [47], increasing evidences suggested that protein complexity derived from alternative splicing play a vital role in cancer progression [13, 48, 49]. AS may induce the insertion, deletion or substitution of functional domains in proteins, resulting in alteration of cellular function [50]. To further predict the potential functional elements in coding proteins that are involved in glioma patient prognosis, we aligned the former data on IDH status-related splicing events with data on canonical protein products of genes in UniProt (www.uniprot.org), which contains annotations of known domains.

Zinc finger proteins (ZNFs), which contain ZNF domains, are an extensive family of proteins that contribute to varied biological functions, including cancer progression [51]. Cys2His2(C2H2) was the first discovered and best characterized type of ZNF and has various functions, including sequence-specific DNA-binding and RNA binding [52]. AS of ZNFs was observed to be related to IDH status. Moreover, the type of AS was basically AT, and the alternative exons were ZNF domains, namely, the alteration of deletion of the ZNF domains at the 3′ end correlated with IDH status. From the analysis results, a higher proportion of the deletions of the zinc finger motif in ZNFs (ZNF283, ZNF724P, ZSCAN20, ZNF606, ZNF169, ZNF430, ZNF20, KDM2B, etc.) were found in samples with IDH mutations (Fig. 6b, c), with similar results in pST2–7 samples (Fig. 4a), demonstrating that zinc fingers might contribute to malignant progression in glioma. Moreover, most of the alternative exons in these ZNFs encode tandem C2H2-type zinc finger motifs, except for KDM2B, which harbors CXXC-type and plant homeodomain (PHD)-type zinc finger motifs.

## Discussion

This study presents the first clustering analysis of prognosis-related AS in glioma. In contrast to previous glioma classification analysis of genomics, RNA expression profiling, epigenetics or proteomics [1, 2, 53], our study focused on AS, a biological process that affects the diversity of protein isoforms and functions.

Survival-related splicing events were identified systematically in pan-glioma and GBM samples from TCGA. A remarkably higher proportion of prognostic splicing events was observed across pan-glioma samples than in GBM samples. In addition, our data revealed clear differences between GBM and LGG samples. We conducted a clustering analysis of the merged GBM and LGG datasets and identified a total of 7 groups. The strong association between glioma grade (GBM versus LGG) and pan-glioma splicing classification that was observed in this study was in accordance with clustering based on proteomics [2]. These results indicated that AS, as a co- or posttranscriptional process that affects protein translation, might contribute to glioma prognosis through influencing the histological phenotype and tumor grade.

As the only GBM-like cluster, pST1 had unique splicing characteristics compared to the remaining 6 LGG-like clusters, providing new methods to distinguish glioma grade. Furthermore, another significant finding of this study is that we identified pST2, an LGG cluster that harbored malignant phenotypes similar to those of GBM, predominantly IDH-wild type, indicating a novel method to distinguish low-grade gliomas with unfavorable prognoses based on iconic splicing events. The cluster-specific study also provided a new method to classify gliomas with different phenotypes using the detection of AS. Due to the limited samples in this study, larger cohorts are needed for verification.

Using the same strategy, the GBM cohort was separated into 3 STs. Among these types, ST1 was identified as the most distinctive subtype with the most unfavorable prognosis and was associated with recognized malignant phenotypic molecular characteristics. A previous GBM molecular classification study found that the proneural class is related to better outcomes but does not benefit from aggressive treatment [1]. Conversely, our study suggested that ST3 had a favorable phenotype, with not only the best prognosis but also the strongest chemoradiotherapy sensitivity, indicating that splicing profiles may be independent of the transcriptome and affect GBM biological behavior. Furthermore, the study suggested that concurrent chemoradiotherapy might be particularly important for improving the prognosis of ST3 patients, but this possibility requires further clinical data.

Clustering of glioblastoma or pan-glioma based on splicing profiles had advantages in identifying clusters with clinically significant prognostic phenotypes, including malignant clusters, such as the ST1 GBM group and the pST2 LGG-like group, and benign phenotypes, such as the ST3 GBM group and the pST7 LGG-like group.

IDH status is considered to be the primary factor in most clustering studies [2], while a recent study revealed a link between IDH status and splicing events [9]. Consistent with this finding, our data also demonstrated that prognostic splicing-based clustering was correlated with IDH status. However, the mechanism that underlies the relationship between IDH status and AS remains unclear. Although previous research revealed the correlation of spliceosome mutations and IDH status in primary myelofibrosis [54, 55], similar results were not observed in glioblastoma [56]. One family that has a remarkable relationship with IDH status is ZNFs, which are also related to tumor grade; more deletions of ZNF domains were detected in IDH-mutant or LGG samples. Evidence showed that disruption of ZNF domains due to AS in ZNFs resulted in dysfunction of transcriptional regulation [57, 58]. Moreover, we found that most of the alteration of deletion of the ZNF domains correlated with IDH status was at the 3′ end, which might introduce premature stop codon (PTC) or nonsense mediated decay (NMD) into mRNA transcript, thereby regulate its stability [59]. We further aligned the former AS analysis with corresponding RNA-seq data. Interestingly, negative relation between deletion of ZNF domain and mRNA expression level in some ZNFs (ZNF283, ZNF724P, ZSCAN20, ZNF606, ZNF20, etc.) were observed (Additional file 11: Figure S5), which might indicate this kind of AS introduced PTC or NMD and caused decay into mRNA transcript. However, this phenomenon was not universal in all ZNFs, demonstrating the underlying mechanism of deletion of ZNF domain in glioma was complicated and needed further investigation. Studies on the target genes of these alternative ZNF domains might provide insights into the significance of AS of ZNFs in glioma. Further studies that block the interaction between ZNFs and their target genes may reveal novel strategies to overcome glioma.

Regulatory network analysis of splicing factors and prognostic splicing events revealed a strong correlation between these two factors in glioma. PTBP1 and HNRNPs [13–15, 40] are recognized as important splicing factors in glioma progression that regulate various splicing events, which is also reflected in our analysis. We also found SNRPB and CELF2 as hub splicing factors of prognostic splicing events, indicating that these splicing factors might be potential targets for glioma treatment. Further research on the biological functions of these splicing factors and their downstream splicing events is needed.

## Conclusion

In this study, a comprehensive analysis of AS in glioma was conducted. Novel classification of glioma based on AS profiles was established, which was associated with glioma grade. Regulatory network of splicing factors and prognostic splicing events were shown; suggesting hub splicing factors including SNRPB and CELF2 were participating in splicing regulatory in glioma. Moreover, we also identified IDH-status-related splicing events profiles. This study might shed new light on glioma heterogeneity and provide new insights into glioma diagnosis and treatment.

## Supplementary information

**Additional file 1: Table S1.** Prognostic Splicing Events in Pan-glioma.

**Additional file 2: Table S2.** Prognostic Splicing Events in GBM.

**Additional file 3: Table S3.** Specific Splicing Events of pST7.

**Additional file 4: Table S4.** Specific Splicing Events of ST3.

**Additional file 5: Table S5.** Correlation between Splicing Factors and pST1-specific Splicing Events (coeffcient > 0.45 or < − 0.45).

**Additional file 6: Table S6.** IDH-status-related splicing events.

**Additional file 7: Figure S1.** Related to Fig. 2. **A** P-values for k=2 to k=8. **B** PAC score curve for k=2 to k=8. **C** RCSI curve for k=2 to k=8.

**Additional file 8: Figure S2.** Related to Fig. 3. **A** PAC score curve for k=2 to k=8. **B** RCSI curve for k=2 to k=8.

**Additional file 9: Figure S3.** DNA methylation among glioma splicing types. **A** DNA methylation among pST1-7. **B** DNA methylation among ST1-3.

**Additional file 10: Figure S4.** Relative expression level of 22 splicing factors in pST1-7 glioma samples.

**Additional file 11: Figure S5.** Scatter diagrams of PSI values of zinc finger proteins alternative splicing events and related gene expression levels.

## Data Availability

RNA-seq data and corresponding clinical data for 665 glioma samples (154 GBM, 511 LGG) were acquired from the data portal for TCGA (https://portal.gdc.cancer.gov/;DbGaP Study Accession:phs000178), using R/Bioconductor package TCGAbiolinks, which are publicly available. Percent Spliced In (PSI) values, the percentage of splicing events in the abovementioned samples, were downloaded by using TCGASpliceSeq (http://bioinformatics.mdanderson.org/TCGASpliceSeq), a publicly available web-based platform that provides splicing patterns of TCGA tumors.

## Abbreviations

AA: Alternate Acceptor site
AD: Alternate Donor
AP: Alternate Promoter
AS: Alternative splicing
AT: Alternate Terminator
CDF: Cumulative distribution function
CIMP: CpG island methylator phenotype
ES: Exon skip
GBM: Glioblastoma
LGG: Low-grade glioma
M3C: Monte Carlo Consensus Clustering
ME: Mutually Exclusive Exons
PAC: Proportion of ambiguous clustering
PSI: Percent Spliced In
RCSI: Relative cluster stability index
RI: Retained Intron
TCGA: The Cancer Genome Atlas
TMZ: Temozolomide
ZNF: Zinc finger
