# Supplementary figures and images for: Classification of glioma based on prognostic alternative splicing
Source: BMC Med Genomics. 2019 Nov 15;12:165. doi: 10.1186/s12920-019-0603-7 (PMC6858651; doi:10.1186/s12920-019-0603-7)

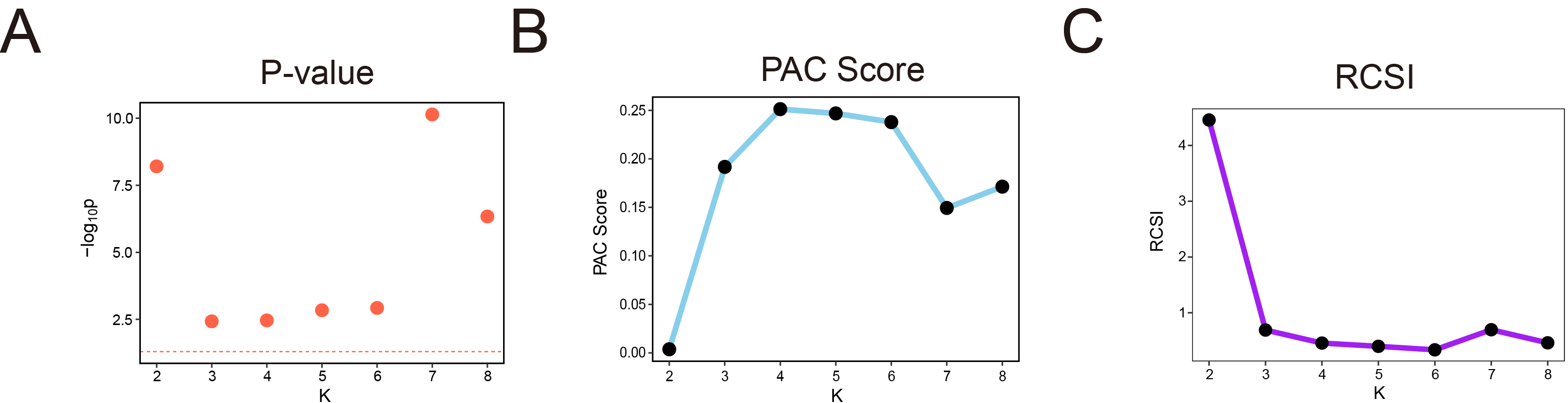

Supplement: Supplementary file 7 — Additional file 7: Figure S1. Related to Fig. 2. A P-values for k=2 to k=8. B PAC score curve for k=2 to k=8. C RCSI curve for k=2 to k=8. [file 12920_2019_603_MOESM7_ESM.tif]

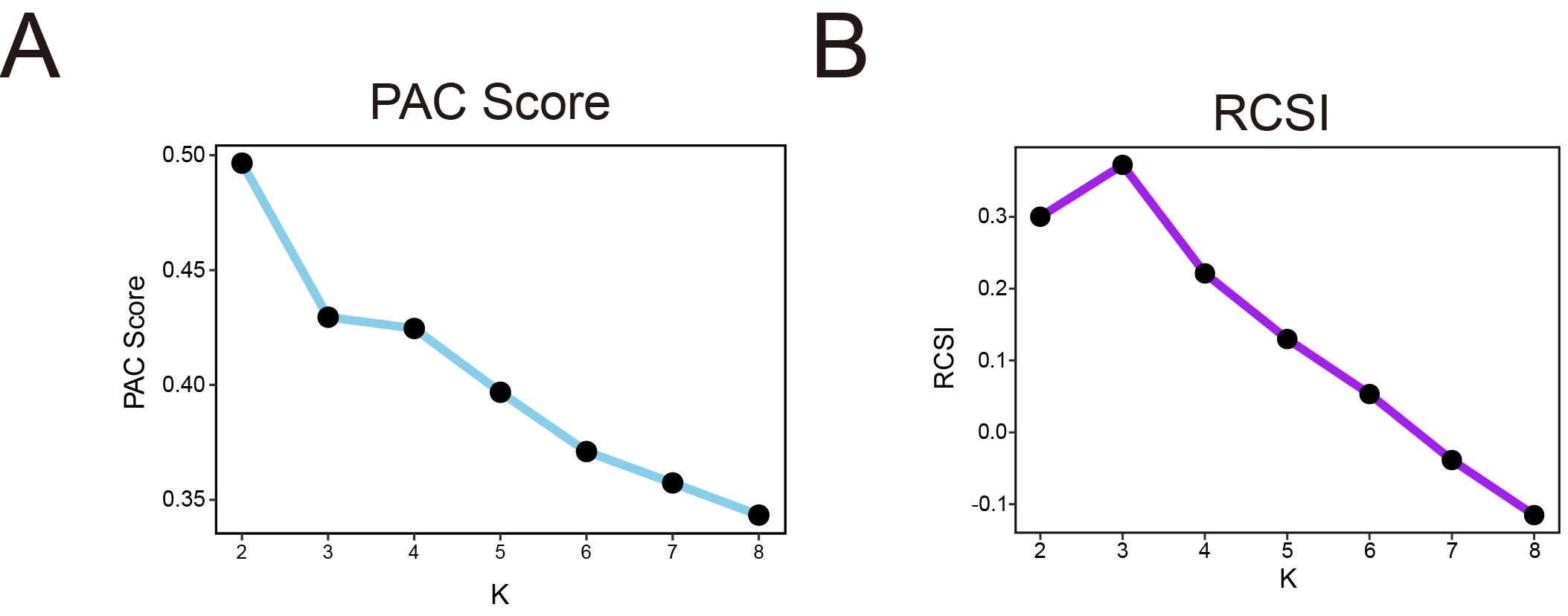

Supplement: Supplementary file 8 — Additional file 8: Figure S2. Related to Fig. 3. A PAC score curve for k=2 to k=8. B RCSI curve for k=2 to k=8. [file 12920_2019_603_MOESM8_ESM.tif]

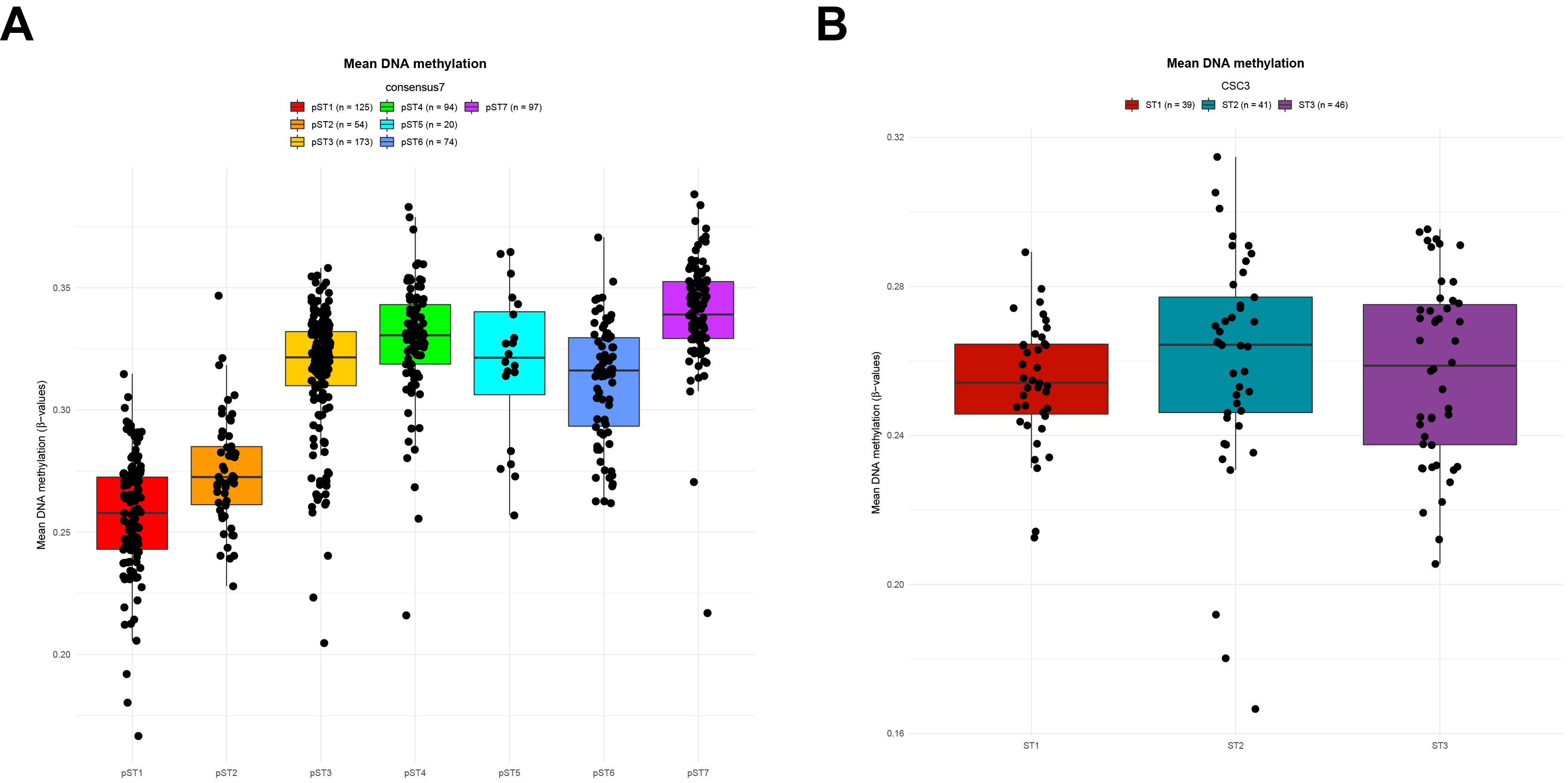

Supplement: Supplementary file 9 — Additional file 9: Figure S3. DNA methylation among glioma splicing types. A DNA methylation among pST1-7. B DNA methylation among ST1-3. [file 12920_2019_603_MOESM9_ESM.tif]
